# Glutathione Stimulates Vitamin D Regulatory and Glucose-Metabolism Genes, Lowers Oxidative Stress and Inflammation, and Increases 25-Hydroxy-Vitamin D Levels in Blood: A Novel Approach to Treat 25-Hydroxyvitamin D Deficiency

**DOI:** 10.1089/ars.2017.7462

**Published:** 2018-10-24

**Authors:** Sushil K. Jain, Rajesh Parsanathan, Arunkumar E. Achari, Preeti Kanikarla-Marie, Joseph A. Bocchini

**Affiliations:** Department of Pediatrics, Louisiana State University Health Sciences Center, Shreveport, Louisiana.

**Keywords:** glutathione, 25-hydroxyvitamin D, vitamin D binding protein, vitamin D receptor, inflammation, insulin resistance

## Abstract

***Aims:*** 25-Hydroxyvitamin D [25(OH)VD] deficiency/inadequacy is a major public health issue affecting more than 1 billion people worldwide. A convincing association exists between low levels of circulating 25(OH)VD and the poor health outcomes associated with chronic diseases. However, high supraphysiological doses of VD are needed to achieve the required 25(OH)VD levels in the blood, because many subjects respond poorly to supplementation.

***Results:*** This study reports a link between 25(OH)VD deficiency and a reduction in glutathione (GSH) in obese adolescents. The improvement in GSH status that results from cosupplementation with VD and l-cysteine (LC; a GSH precursor) significantly reduced oxidative stress in a mouse model of 25(OH)VD deficiency. It also positively upregulated VD regulatory genes (*VDBP*/VD-25-hydroxylase/*VDR*) in the liver and glucose metabolism genes *(PGC-1α/VDR/GLUT-4)* in muscle, boosted 25(OH)VD, and reduced inflammation and insulin resistance (IR) levels in the blood compared with supplementation with VD alone. *In vitro* GSH deficiency caused increased oxidative stress and downregulation of *VDBP*/VD-25-hydroxylase/*VDR* and upregulation of *CYP24a1* in hepatocytes and downregulation of *PGC-1α/VDR/GLUT-4* in myotubes. This study demonstrates that improvement in the GSH status exerts beneficial effects on the blood levels of 25(OH)VD, as well as on the inflammation and IR in a VD-deficient mouse model. Thus, the VD supplements widely consumed by the public are unlikely to be successful unless the GSH status is also corrected.

***Innovation:*** These studies demonstrate a previously undiscovered mechanism by which GSH status positively upregulates the bioavailability of 25(OH)VD.

***Conclusion:*** Supplementation with a combination of VD and LC or GSH precursor, rather than supplementation with VD alone, is beneficial and helps achieve more successful VD supplementation.

## Introduction

Changes in modern lifestyles that limit physical and outdoor activity and increased consumption of high-energy diets have led to a high incidence of obesity and diabetes and widespread inadequacy/deficiency of 25-hydroxyvitamin D [25(OH)VD] in populations worldwide. Epidemiological studies provide conclusive evidence that lower circulating levels of 25(OH)VD are associated with the poor outcomes frequently associated with several chronic metabolic diseases (7, 45, 56). This has led to widespread use of vitamin D (VD) supplements by the public attempting to achieve better health (21, 34, 41). However, randomized controlled clinical trials have shown that high supraphysiological doses of VD are needed to achieve the required levels of VD in the circulation and that not all subjects respond to vitamin D (VD) supplementation (22, 31, 33, 51).

Innovation The status of 25-hydroxyvitamin D [25(OH)VD] plays a crucial role in preventing disease and maintaining optimal health. These studies demonstrate a previously undiscovered mechanism by which increasing glutathione (GSH) status positively influences the bioavailability of 25(OH)VD. The improvement in GSH caused by supplementation with vitamin D (VD) combined with l-cysteine significantly upregulates expression of VD regulatory genes and glucose metabolism genes, and provides a novel approach to correct the widespread 25(OH)VD inadequacy/deficiency found in populations worldwide.

Risk factors for 25(OH)VD deficiencies include race (darker pigmented skin tones), higher body mass index (BMI), winter season, higher geographic latitudes, and diet. Circulating 25(OH)VD is considered to be a comprehensive and stable metabolite, levels of which can be used to diagnose 25(OH)VD deficiency and monitor VD consumption (8). The metabolic factors responsible for the limited success of VD supplementation studies, despite the convincing association between low 25(OH)VD levels and poor health, remain unknown.

VD or cholecalciferol in the human body is derived mostly from either diet or from skin exposure to ultraviolet B from sunlight (39, 45, 49, 59). Most people require dietary supplementation with VD to achieve the recommended blood levels of 25(OH)VD. The liver is the principal site where cholecalciferol is converted to 25(OH)VD by VD-25-hydroxylase (cytochrome p450 enzymes [CYP], *CYP2R1, CYP27A1*) (9, 14, 45, 56). 25(OH)VD is bound to vitamin D binding protein (*VDBP*) and transported into the circulation. VDBP is primarily synthesized and secreted by the liver (14, 55). (*CYP27B1*), which converts 25(OH)VD to its active metabolite [1alpha,25-dihydroxyvitamin D3, 1,25(OH)_2_VD], is present in both renal (major site) and nonrenal tissues (1). Even though renal tissue is considered to be a major site for 1,25(OH)VD formation, recent studies demonstrate expression of (*CYP27B1*) in nonrenal cells and tissues, indicating localized 1,25(OH)_2_VD formation and its tissue-specific paracrine function in different tissues (1).

(*CYP24A1*) is involved in the catabolic inactivation of 1,25(OH)_2_D_3_ and its inhibition is thought to limit 1,25(OH)_2_D_3_ signaling (39). Genetic variations in VDBP/(*CYP2R1*) are known to influence 25(OH)VD blood levels in response to VD supplementation (13, 19, 23, 29, 30, 52). Most cells have receptors for VD known as vitamin D receptor (*VDR*) (60). The biological actions of 1,25(OH)_2_VD are directly related to the VDR content of target tissues. Muscle is a major site of glucose metabolism and maintenance of glucose homeostasis (12). Therefore, biosynthesis and metabolism of VD are under the control of VD regulatory genes (GC/*VDBP*/VD-25-hydroxylase) in the liver, while the downstream actions of the active 1,25(OH)_2_VD in muscle are mediated by glucose metabolism genes (*VDR*/peroxisome proliferator-activated receptor gamma coactivator 1-alpha [*PGC-1*α]/glucose transporter type 4 [*GLUT-4*]).

Glutathione (GSH) is a major antioxidant and its depletion increases oxidative stress and extensive carbonylation of proteins (10, 11, 16–18). Oxidative modification or carbonylation covalently modifies endogenous enzymes and proteins, which can result in the loss of protein function, insulin resistance (IR), and impaired cell function, and play a significant role in the etiology of several human diseases (2, 5, 6, 15, 37, 46, 47). Oral supplementation with GSH or l-cysteine (LC; a GSH precursor) has been successfully used to improve the GSH status in blood and tissues while lowering inflammation and IR in humans and animals (28, 38, 58, 61). However, there is no report in the literature of a link between impaired GSH status and impaired status of the VD regulatory genes in the liver or glucose metabolism genes in muscle.

This article discusses our investigation of the dual roles of GSH in increasing circulating 25(OH)VD and augmenting the actions of active VD metabolites in one of the target tissues (skeletal muscle), which is a major site for glucose metabolism.

## Results

### Association between GSH and 25(OH)VD blood levels in adolescents

**FIG. 1.**
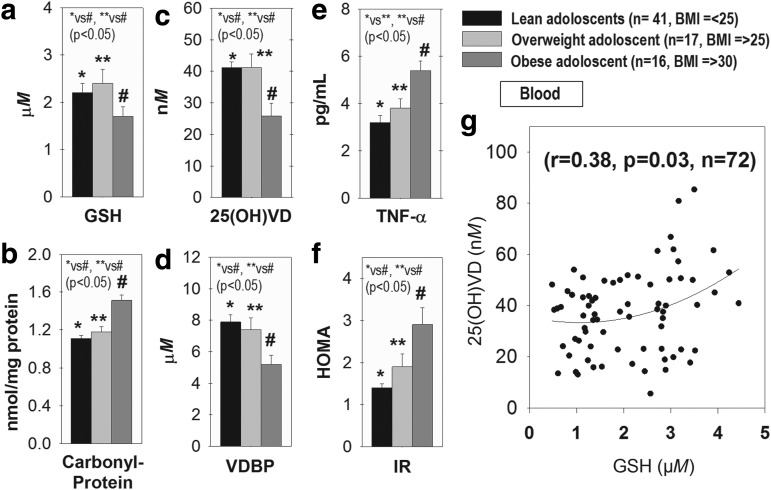
**Blood levels of 25(OH)VD and GSH and its positive association in obese adolescent. **Blood levels of GSH **(a)**, carbonyl-protein **(b)**, 25(OH)VD **(c)**, VDBP **(d)**, TNF-α **(e)**, and HOMA-IR **(f)** in lean, overweight, and obese adolescents. This illustrates a significant reduction in GSH and 25(OH)VD and increase in TNF-α, carbonyl-protein, and IR levels in obese subjects, and that 25(OH)VD levels are positively correlated with the GSH status in adolescents **(g)**. BMI was used as an additional independent variable to calculate *r* and *p*-values for the correlation between 25(OH)VD and GSH. Mean ± SE; data analyzed using one-way ANOVA. 25(OH)VD, 25-hydroxyvitamin D; ANOVA, analysis of variance; BMI, body mass index; GSH, glutathione; HOMA-IR, homeostatic model assessment insulin resistance; IR, insulin resistance; TNF-α, tumor necrosis factor alpha; VDBP, vitamin D binding protein.

Blood levels of GSH and 25(OH)VD were significantly lower in obese compared with lean or overweight adolescents (Fig. 1a, c). VDBP levels were significantly lower in obese children compared with both lean and overweight (Fig. 1d). Blood levels of carbonyl protein were significantly elevated in obese adolescents compared with lean and overweight, suggesting elevated oxidative stress level in obese subjects (Fig. 1b). Tumor necrosis factor alpha (*TNF-α*) levels and homeostatic model assessment (HOMA)-IR were significantly higher in obese compared with lean or overweight subjects (Fig. 1e, f). Figure 1g shows a significant positive correlation between 25(OH)VD and GSH (*r* = 0.38, *p* = 0.03, *n* = 72).

Supplementary Table S1 (Supplementary Data are available online at www.liebertpub.com/ars) shows that 25(OH)VD has a negative association with IR (*r* = −0.28, *p* = 0.04); IR also showed a negative correlation with GSH (*r* = −0.25, *p* = 0.05) and a positive association with *TNF-α* (*r* = 0.27, *p* = 0.04). BMI shows negative association with 25(OH)VD (*r* = −0.25) and positive association with TNF-α (*r* = 0.39). TNF-α showed positive association with BMI (*r* = 0.39), protein carbonyl (*r* = 0.37), and IR (*r* = 0.29). Protein carbonyl association with GSH was not significant. Ages of subjects in each group were similar, while BMI levels were significantly different in each group. Subject enrollment detail is given previously (43). Studies demonstrate low plasma levels of 25(OH)VD in humans with genetic mutation for *VDBP* or in VDBP-knockdown (KD) mouse models (19, 30, 52). Blood concentrations of VDBP are positively related to the half-life of circulating 25(OH)VD (30).

This suggests that lower *VDBP* can contribute to decreased circulating 25(OH)VD levels in obese adolescents. A positive association exists between blood levels of GSH and those of 25(OH)VD and has been previously shown in adult diabetic patients (3, 25, 27). The present finding of a positive association between circulating 25(OH)VD and GSH status is unique and interesting because in contrast to adults, the adolescent population has a narrow age range (14–17 years) and does not have any of the confounding variables such as medications or clinical disorder. This led us to search whether GSH regulates VD regulatory genes and 25(OH)VD status, and additionally whether GSH deficiency increases *TNF-α* levels and downregulates *PGC-1α/VDR/GLUT-4* signaling of glucose metabolism.

### Effect of high-fat diet feeding on GSH, 25(OH)VD, and carbonyl protein levels in blood, and GSH metabolism genes and VD regulatory genes in liver and muscle in mice

**FIG. 2.**
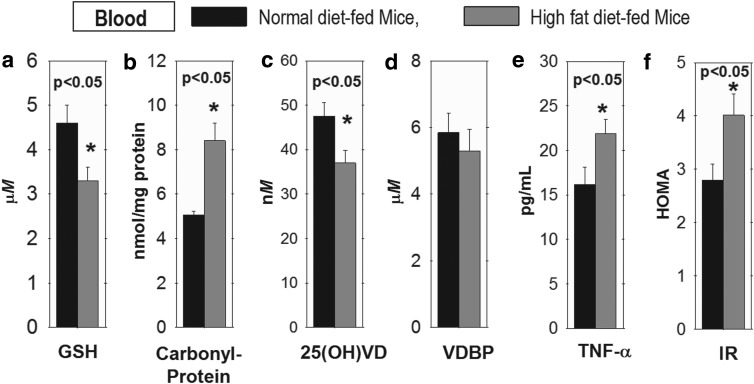
**This figure illustrates a significant decrease in GSH and 25(OH)VD, and significantly increased carbonyl protein, TNF-α, and IR levels in the blood of HFD-fed mice compared with those of mice fed with control diet.** **(a)** GSH, **(b)** Carbonyl protein, **(c)** 25(OH)VD, **(d)** VDBP, **(e)** TNF-α, **(f)** IR in the blood of HFD-fed mice compared to control group. Mean ± SE (*n* = 7); data analyzed using unpaired Student's *t*-test. HFD, high-fat diet.

Male C57BL/6J mice (5 weeks old) were purchased from The Jackson Laboratory (Bar Harbor, ME). The animals were fed either a standard chow diet (Control: Harlan TD.08485, providing 5.2% calories as fat) or a high-fat diet (HFD) for 16 weeks (36). Composition of normal and HFD is given previously (36). Data given in Figure 2 show that the HFD-fed mice exhibited significantly lower levels of GSH (Fig. 2a) and 25(OH)VD (Fig. 2c) and higher levels of carbonyl protein (Fig. 2b), TNF-α (Fig. 2e), and IR (Fig. 2f) similar to the obese adolescent subjects' data. VDBP levels were not significantly different between HFD- *versus* control diet-fed group. Body weight (BW), food intake, parathyroid hormone (PTH), calcium, and blood count levels in the blood of mice fed normal diet and HFD mice groups were similar (Supplementary Table S2).

**FIG. 3.**
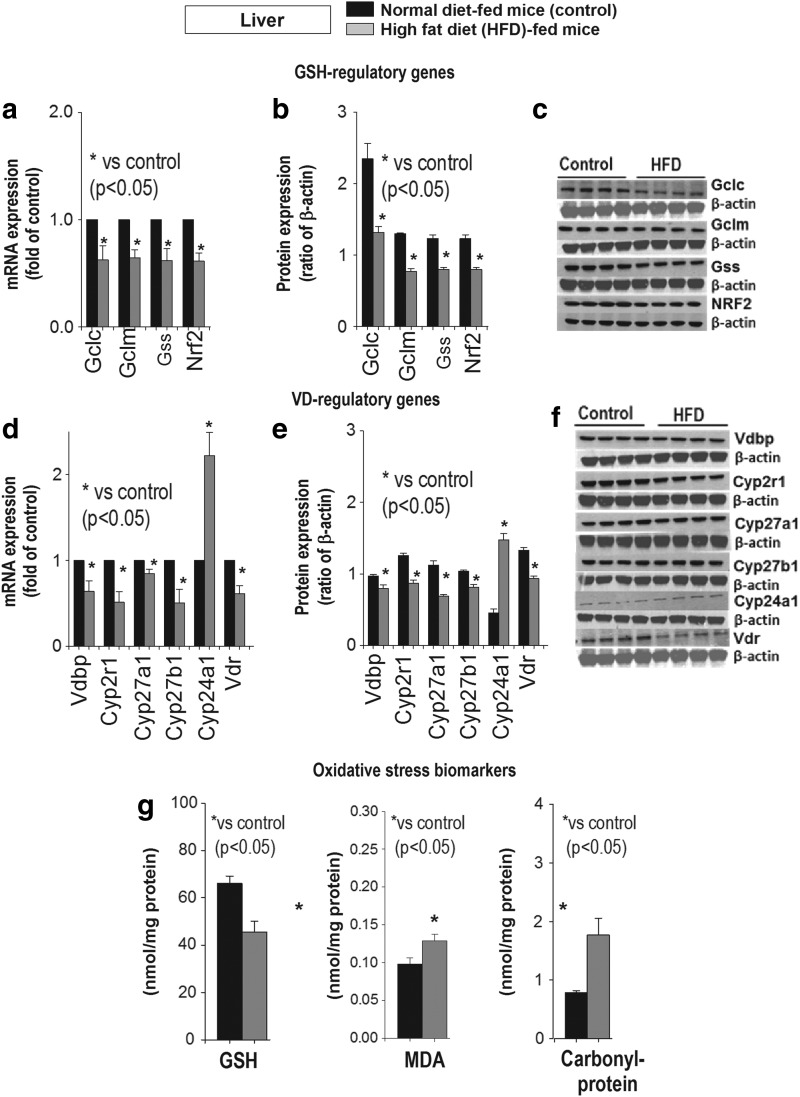
**Effect of HFD or control diet on mRNA and protein expression of GSH regulating genes (a–c), VD regulating genes (d–f), and oxidative stress biomarker levels (g) in liver of mice.** HFD caused significant downregulation of VD regulatory, reduced GSH, and increases in oxidative stress. Mean ± SE (*n* = 7); data analyzed using unpaired Student's *t*-test. GCLC, glutamate-cysteine ligase catalytic subunit; GCLM, glutamate-custeine ligase regulatory subunit; VD, vitamin D; VDBP, vitamin D binding protein; VDR, vitamin D receptor.

Figure 3 shows the mRNA and protein expression of genes *GCLC* (glutamate-cystein ligase catalytic subunit)/*GCLM* (glutamate-cysteine ligase regulatory subunit)/*GSS/NRF2* (nuclear factor erythroid-2-related factor) involved in GSH synthesis (Fig. 3a–c), and genes *VDBP/CYP2R1/CYP27B1/VDR*, which determine bioavailability of 25(OH)VD, were significantly downregulated (Fig. 3d–f) in livers of HFD-fed mice compared with normal diet-fed mice. Interestingly, mRNA and protein expression levels of *CYP24A1* that degrade 25(OH)VD are upregulated in the liver of mice fed HFD in comparison with mice fed normal diet.

**FIG. 4.**
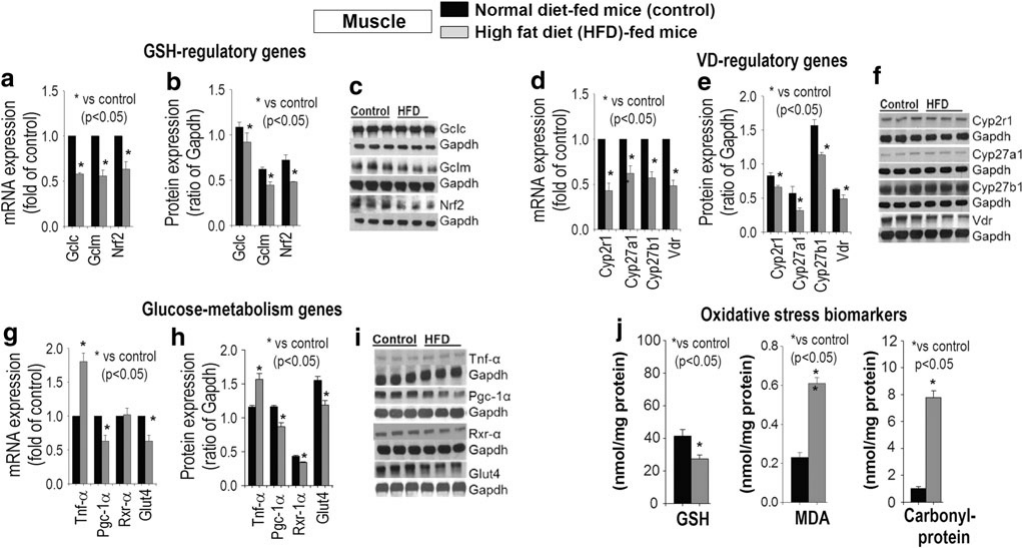
**Effect of HFD or control diet on mRNA and protein expression of GSH regulating genes (a–c), VD regulating genes (d–f), glucose metabolism genes (g–i), and oxidative stress biomarker levels (j) in skeletal muscle of mice.** HFD caused significant downregulation of VD regulatory and GSH biosynthesis genes, reduced GSH, and increases in carbonyl protein and MDA levels in the skeletal muscle; and a downregulation of *PGC-1α/GLUT-4* and upregulation of *TNF-α* in comparison with mice fed a control diet. Mean ± SE (*n* = 7); data analyzed using unpaired Student's *t*-test. GLUT-4, glucose transporter type 4; MDA, malondialdehyde; PGC-1α, peroxisome proliferator-activated receptor gamma coactivator 1-alpha; RXRα, retinoic X receptor.

Figure 4 shows a significant decrease in total GSH and increased oxidative stress markers (Fig. 4j) in muscle. GSH (*GCLC/GCLM/NRF2*) and VD regulatory genes (*VDBP/CYP2R1/CYP27B1*) are downregulated significantly (Fig. 4a–f) in HFD-fed mice muscle. Figure 4g–i shows that mRNA and protein expression of genes that regulate glucose metabolism (*VDR/PGC-1α/GLUT-4*) is significantly downregulated and TNF-α increased in the muscle of mice fed HFD compared with normal diet-fed mice group.

There was a significant increase in lipid peroxidation and protein oxidation with decreased GSH levels in skeletal muscle (Fig. 4j) of HFD-fed compared with normal diet-fed mice. A similar trend was observed in liver tissue (Fig. 3g). Protein-bound carbonyls are relatively more stable than lipid peroxidation products (10, 11). This demonstrates that HFD consumption increases cellular oxidative stress levels.

Overall, HFD feeding resulted in a significant downregulation of genes that synthesize GSH and lower levels of GSH in blood, liver, and muscle. Similarly, there was a significant downregulation of VD regulatory genes in the liver and muscle of mice fed HFD. In addition, there was a significant downregulation of glucose metabolism genes in muscle of mice fed HFD in comparison with normal diet-fed mice group. A decrease in blood and tissue GSH reflects exhaustion or impaired antioxidant pathways and increased oxidative stress in tissues in mice consuming HFD.

### Effect of supplementation with VD along with LC on plasma levels of GSH, 25(OH)VD, and IR, and on GSH and VD regulatory genes in liver and on GSH and glucose metabolism genes in muscle

**FIG. 5.**
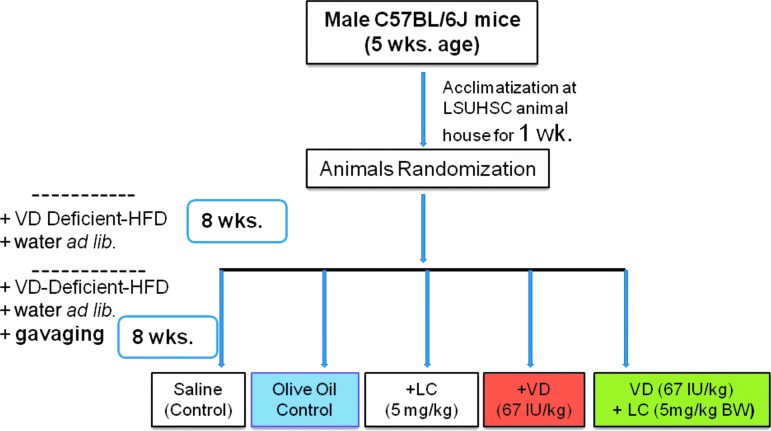
**Experimental design for supplementation of placebo (saline,** ***n* = 7), OO—control, LC (*n* = 6), VD (*n* = 6), and combined VD+LC (*n* = 6).** Mice were purchased at 5 weeks of age and then kept in institutional animal house for acclimatization for 1 week. Mice were then randomized into different groups and were maintained on VD-deficient HFD (to mimic VD deficiency) for 8 weeks. Then mice were also gavaged with saline, LC, OO, VD, or VD+LC for 8 weeks. VD was dissolved in OO and one group of mice also gavaged with similar amounts of OO (vehicle alone). VD *versus* VD+LC groups have similar amounts of VD and OO-vehicle. BW, body weight; LC, L-cysteine; OO, olive oil.

Beginning at 5 weeks of age, male C57BL/6J mice were fed and maintained on a VD-deficient HFD and water *ad libitum* for 16 weeks (36). Mice were gavaged daily for the last 8 weeks with saline, olive oil (OO), LC (5 mg/kg BW), VD (67 IU/kg BW), or LC+VD to investigate whether cosupplementation with GSH precursor has a better effect on blood levels of 25(OH)VD compared with levels achieved using VD alone. VD was dissolved in OO and one group of mice was also gavaged with OO (vehicle) alone. Details of experimental design are shown in Figure 5.

**FIG. 6.**
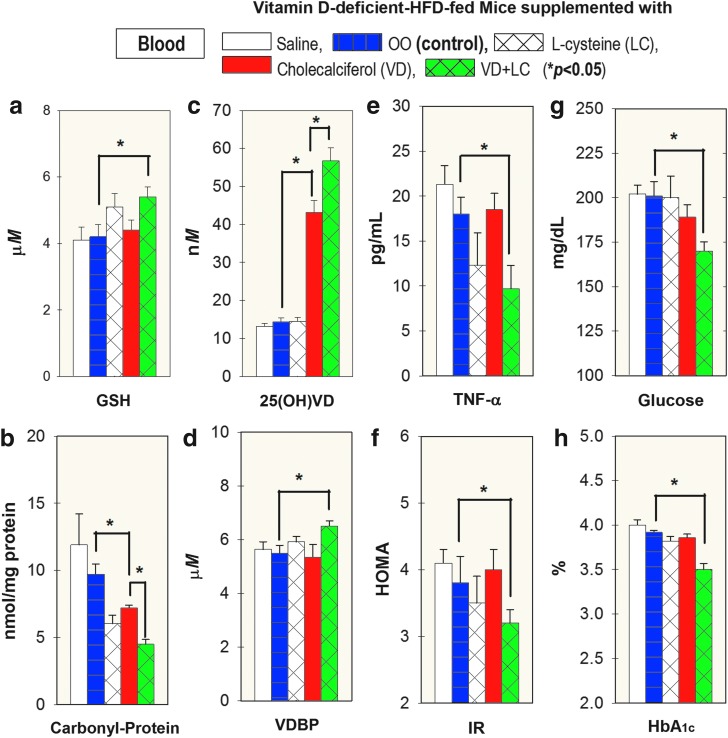
**Effect of supplementation with VD+LC (*green bar*)** ***versus*** **VD alone (*red bar*) on blood levels of GSH (a), carbonyl protein (b), 25 (OH)VD (c), VDBP (d), TNF-α (e), HOMA-IR (f), fasting glucose (g), and HbA_1c_ (h) in mice maintained on a VD-deficient HFD for 16 weeks.** Mice were gavaged with saline, OO, LC, VD, or VD+LC during last 8 weeks. VD was dissolved in OO and one group was also gavaged with OO (vehicle) alone. This shows a significantly greater increase in GSH and 25(OH)VD, and lower TNF-α, IR, glucose, and HbA_1c_ levels in combined VD+LC compared with those supplemented with VD alone. Mean ± SE (*n* = 6); data analyzed using ANOVA Holm–Sidak method with vehicle (OO) group as a control (*blue bar*).

The effect of VD with and without cosupplementation with LC on blood levels of GSH, carbonyl protein, 25(OH)VD, VDBP, *TNF-α*, IR, glucose, and HbA_1c_ is shown in Figure 6a–h. VD alone did not show effect on GSH, *TNF-α, VDBP*, IR, and HbA_1c_. However, supplementation with combined VD (cholecalciferol)+LC significantly corrected the GSH status and showed a significant decrease in *TNF-α*, IR, and HbA_1c_ in the blood compared with levels seen in the mice supplemented with OO (vehicle)-control group. In addition, supplementation with combined VD (cholecalciferol)+LC showed a greater increase in 25(OH)VD and decrease in protein carbonylation levels in the blood compared with the OO-supplemented control mice. There were no changes in food intake, RBC indices, or calcium among these groups. There was also no change in blood counts, which indicates that the decrease in HbA_1c_ values seen in the VD+LC group was not due to any effect on cell viability (Supplementary Table S3).

**FIG. 7.**
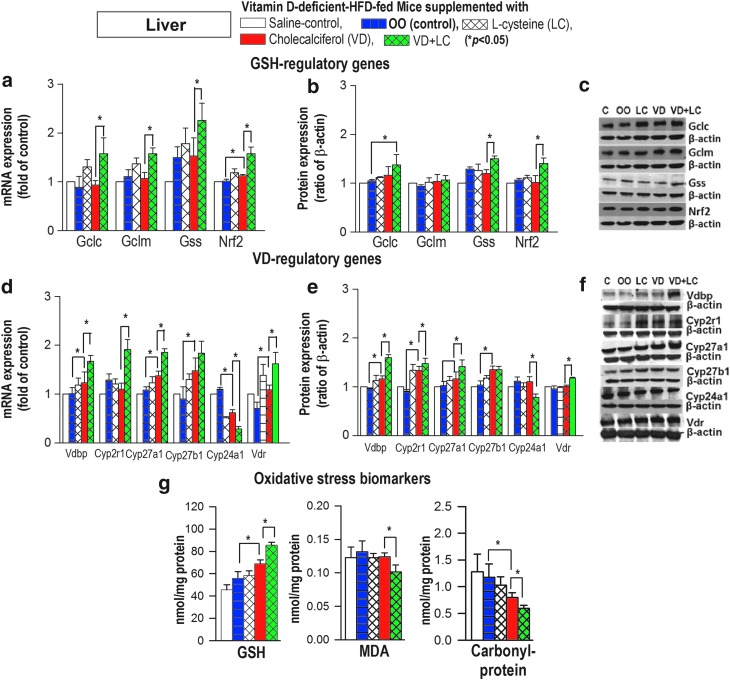
**Effect of supplementation with VD+LC (*green bar*)** ***versus*** **VD alone (*red bar*) on mRNA and protein expression of GSH biosynthesis genes (a–c), VD regulating genes (d–f), and oxidative stress biomarkers (g) in livers of mice maintained on a VD-deficient HFD for 16 weeks.** Mice were gavaged with saline, OO, LC, VD, or VD+LC during last 8 weeks. Compared with VD alone, combined VD+LC showed a significantly greater upregulation of VD regulatory and GSH biosynthesis genes, increased GSH, and lower oxidative stress. Mean ± SE (*n* = 6) and analyzed using ANOVA Holm–Sidak method with vehicle (OO) group as a control (*blue bar*).

**FIG. 8.**
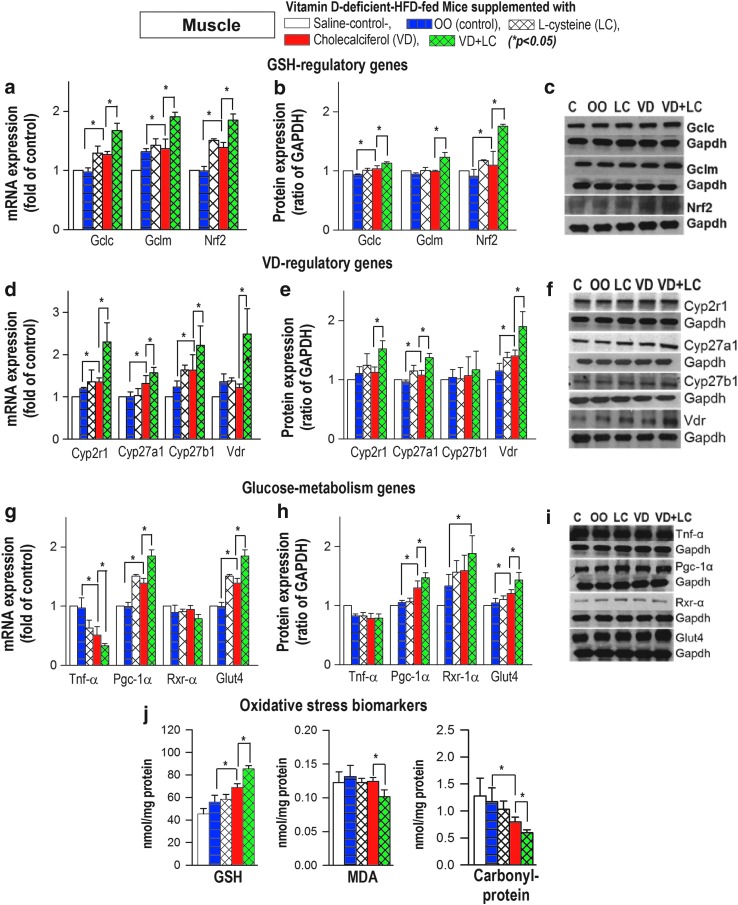
**Effect of oral supplementation with VD+LC (*green bar*)** ***versus*** **VD alone (*red bar*) on mRNA and protein expression of GSH biosynthesis genes (a–c), VD regulating genes (d–f), glucose metabolism genes (g–i), and oxidative stress biomarkers (j) in skeletal muscle of mice maintained on VD-deficient HFD for 16 weeks.** Compared with VD alone, combined VD+LC showed a greater upregulation of VD regulatory and GSH biosynthesis genes, increased GSH, reduced oxidative stress, and a greater upregulation of *PGC-1α/GLUT-4*. Mean ± SE (*n* = 6); data analyzed using ANOVA Holm–Sidak method with vehicle (OO) group as a control (*blue bar*).

Cosupplementation with VD+LC caused a significantly greater upregulation of mRNA and protein expression of GSH synthesizing genes (*GCLC/GSS/NRF2*), GSH status, and VD regulatory genes (*CYP2R1/CYP27A1/VDBP/VDR*) in the liver (Fig. 7a–f). In addition, CYP24A1 showed significantly lower mRNA and protein expression levels in the liver of mice supplemented with VD+LC in comparison with VD-alone mice. This study measured total 25(OH)VD status using an enzyme-linked immunosorbent assay (ELISA) kit. Studies in the literature show that 25(OH)VD analyses using either an ELISA kit or the MC/MS approach show a significant correlation. Similarly, Figure 8 shows that there was a significant upregulation of *GCLC/GCLM/NRF2* (Fig. 6a–c) and VD regulatory genes *CYP2R1/CYP27A1/VDR* (Fig. 8d–f) in the muscle of mice supplemented with VD+LC compared with VD alone.

In addition, there was a significant upregulation of *PGC-1α/GLUT-4* (Fig. 8g–i) and GSH status (Fig. 8j) in muscle of mice supplemented with VD+LC in comparison with VD-alone supplemented mice (Fig. 8g–i). In addition, levels of protein oxidation and lipid peroxidation were significantly reduced in the liver (Fig. 7g) and muscle (Fig. 8j) of mice supplemented with VD+LC compared with those in mice supplemented with VD alone. The increase in GSH and reduction of oxidative stress biomarkers in blood and tissues reflect reduction of oxidative stress levels in tissues of mice supplemented with VD+LC compared with tissues of mice supplemented with VD alone.

**FIG. 9.**
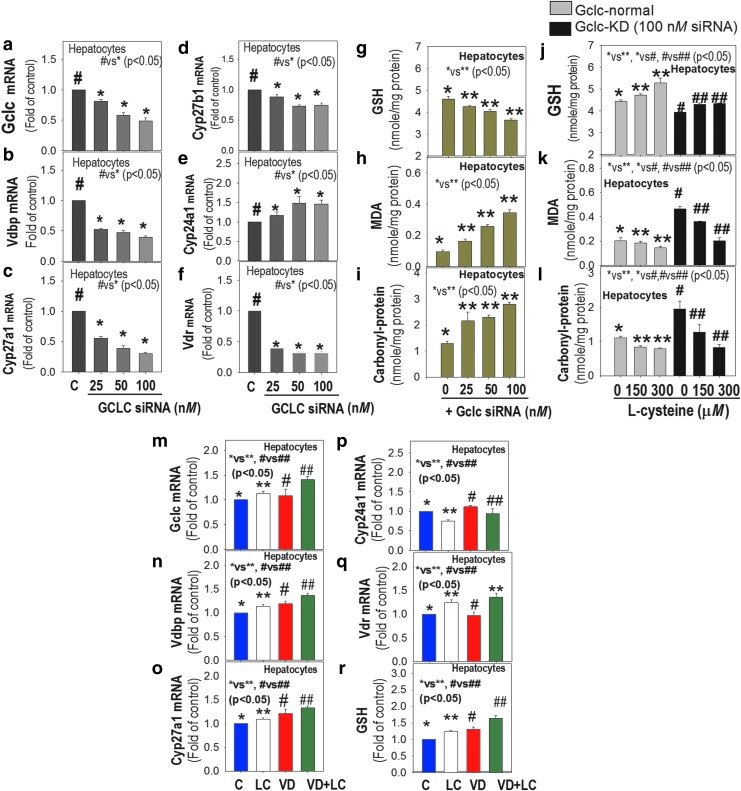
**Effect of GSH-deficiency, L-cysteine, and cholecalciferol supplementation on mouse hepatocytes.** Effect of GCLC-KD on *GCLC* **(a)**, and mRNA levels of VD regulatory genes **(b–f)**. GSH deficiency dose dependently downregulated Vdbp, Cyp27a1, Cyp27b1, and Vdr, and upregulated Cyp24a1. This figure also shows that GSH deficiency decreased GSH **(g)** and increased MDA **(h)** and carbonyl-protein **(i)**; and LC (6 h) increased GSH **(j)** and lowered MDA **(k)** and carbonyl-protein **(l)** in *GCLC*-normal and *GCLC*-KD hepatocytes. Effect of combined LC (300 μ*M*, 2 h preincubation) and VD (10 n*M*, 22 h) on GSH **(r)** and mRNA levels of GCLC **(m)**, Cyp24a1 **(p)**, VDR **(q)**, CYP27A1 **(o)**, and VDBP **(n)** shows that LC increases GSH and upregulation of *CYP27A1/CYP27B1/VDBP* by VD. Mean ± SE (*n* = 3); data analyzed using one-way ANOVA. CYP, cytochrome P450 enzymes; GCLC-KD, glutamate-cysteine ligase catalytic subunit knockdown; VDR, vitamin D receptor.

Figure 9 shows that *GCLC* KD resulted in a dose-dependent decrease in GCLC (Fig. 9a) with downregulation of *VDBP, CYP27A1, CYP27B1,* and *VDR* and upregulation of *CYP24A1* mRNA levels (Fig. 9b–f) in hepatocytes. Figure 9g shows a decrease in the level of GSH and increase in lipid peroxidation and carbonylated protein (Fig. 9h, i) levels in GCLC-KD hepatocytes. The effect of GSH deficiency and of LC supplementation on GSH, malondialdehyde (MDA), and carbonyl protein in GCLC-normal and GCLC-KD hepatocytes is shown in Figure 9j–l. GSH deficiency (8–21%) resulted in significantly increased levels of carbonyl protein (77–277%) and MDA (67–12%) levels, and LC supplementation improved the status of GSH and reduced oxidation of both proteins and lipids in GSH-normal and GSH-deficient hepatocytes. This suggests that improved GSH status has the potential to prevent oxidative stress and mediate the upregulation of VD regulatory gene levels. Details of treatments with GCLC siRNA, LC, and mRNA analyses are given previously (26).

The stimulatory effect of cholecalciferol on VD regulatory genes was higher in LC cosupplemented cells, as shown in Figure 9m–r. GSH deficiency impairs VD regulatory gene expression, whereas cosupplementation with VD and LC can positively modify the status of GSH, *CYP24A1*, and VD regulatory genes in hepatocytes. These studies show that GSH status positively upregulates the gene expression of *CYP27B1* that converts 25(OH)VD to 1,25(OH)_2_VD. *VDR*-KD was induced using siRNA and was able to achieve nearly 80% *VDR*-KD in hepatocytes. *VDR*-KD caused a simultaneous decrease in VD metabolism genes *CYP27A1*, *VDBP*, and *VDR*, while upregulation of genes by VD+LC was abolished in *VDR*-KD hepatocytes (Supplementary Fig. S1). Supplementation with LC alone caused upregulation of *VDBP* but not of the *CYP27A1* gene compared with results in control *VDR*-KD cells (Supplementary Fig. S1). This suggests that VDR mediates the beneficial effect of cholecalciferol on VD metabolism gene upregulation.

**FIG. 10.**
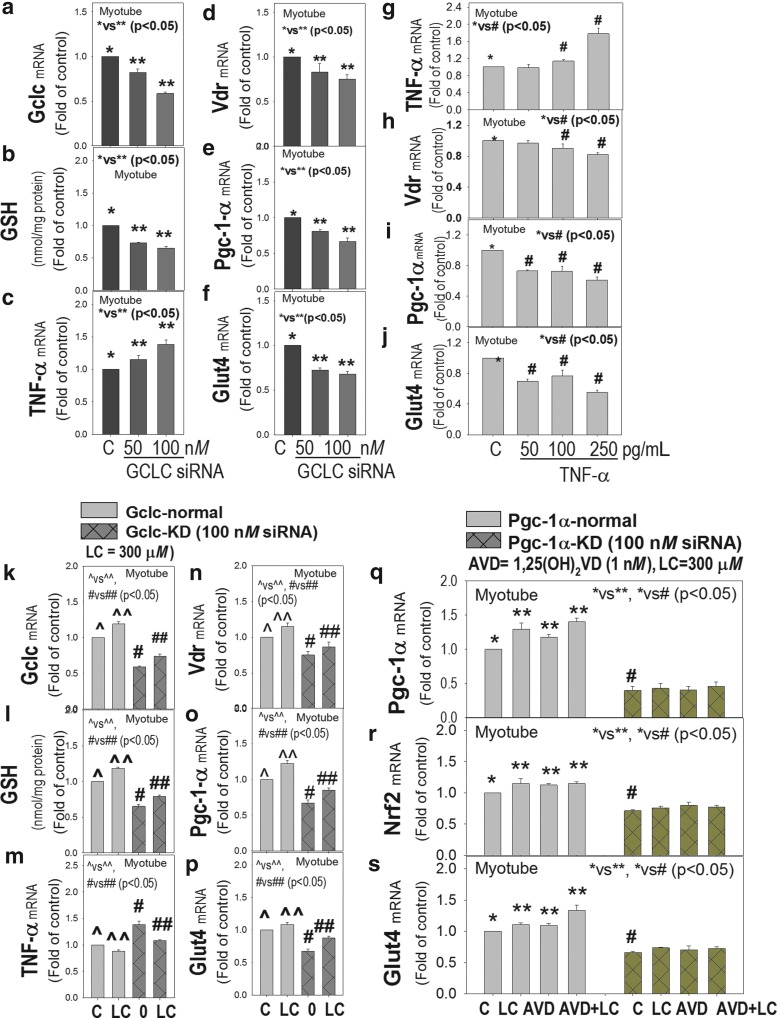
**Effect of GSH-deficiency, TNF-α treatment, L-cysteine, and active vitamin D supplementation on mouse myotubes**. Effect of GCLC-KD **(a–f)** and LC (300 μ*M*, 6 h) **(k, m–p)** on mRNA expression of *GCLC*, *TNF-α*, *VDR*, *PGC-1α*, *GLUT-4*, and GSH in myotubes. GSH deficiency caused a significant downregulation of *VDR*, *PGC-1α*, and *GLUT-4* and an increase in *TNF-α* in GSH-deficient cells. LC helped increase GSH levels and reversed the downregulation of *VDR1α/PGC-1α/GLUT-4* and upregulation of TNF-α **(k–p)**. TNF-α *per se* inhibited the VDR/PGC-1α/GLUT-4 gene expression **(g–j)**. Results **(q–s)** show that the effect of AVD on upregulation of *NRF2* and *GLUT-4* was absent in *PGC-1α* KD myotubes, which indicates that *PGC-1α* mediates the upregulation of *NRF2* and *GLUT-4* by 1,25(OH)_2_VD. Mean ± SE (*n* = 4) and analyzed using one-way ANOVA. AVD, active vitamin D.

Figure 10 shows that GSH deficiency (*GCLC*-KD) caused a simultaneous increase in TNF-α and decrease in *VDR/PGC-1α/GLUT-4* mRNA expression in *GCLC*-KD C2C12 myotubes (Fig. 10a–f). Stimulation with exogenous *TNF-α per se* downregulates *VDR/PGC-1α/GLUT-4* in myotubes (Fig. 10g–j). Supplementation with LC, which increases GSH levels, caused an increase in mRNA expression of *VDR/GLUT-4*, and a decrease in *TNF-α* levels in both *GCLC-*normal and *GCLC*-KD myotubes (Fig. 10k–p). This suggests that GSH deficiency caused an increase in *TNF-α* and decrease in *PGC-1α*, as well as a decrease in the *VDR/GLUT-4* needed for the action of 1,25(OH)_2_VD in muscle. Deficient GSH levels can result in inflammation, which can be reversed by improving GSH status. Further studies examined the effect of combined LC and 1,25(OH)_2_VD and of *PGC-1α* KD on *GLUT-4* in myotubes.

Cotreatment with LC and 1,25(OH)_2_VD (active vitamin D) resulted in significantly greater upregulation of *PGC-1α/NRF2/GLUT-4* (Fig. 10q–s) gene expression in comparison with results from treatment with 1,25(OH)_2_VD alone; however, this was not seen in *PGC-1α*-KD cells. Thus, a reduction in levels of *TNF-α* by LC will result in lowered inflammation and improved glucose metabolism and skeletal muscle function. 1,25(OH)_2_VD was used to understand its efficacy on glucose metabolism genes in muscle cells, whereas cholecalciferol was used with hepatocyte studies to understand 25(OH)VD biosynthesis from cholecalciferol in liver.

## Discussion

GSH is a major antioxidant and a cofactor of many enzymes in the human body (16–18). GSH is readily measured in blood and reflects the *in vivo* defense against oxidative stress (16, 47, 48, 53). This study demonstrates that a reduction in GSH status is linked to 25(OH)VD deficiencies in obese adolescents and in HFD-fed mice. A decrease in blood GSH and increased oxidative stress reflect exhausted or impaired antioxidant pathways in obese humans and HFD-fed mice. Lower levels of GSH can occur because of nonavailability of LC from food consumption, increased ROS production and oxidative stress from energy-rich diet consumption, and/or increased utilization of GSH relative to its biosynthesis. The depletion or deficiency of GSH can increase oxidative stress and extensive carbonylation of proteins, which can increase inflammatory mediators such as TNF-α and impair normal function of endogenous enzymes and proteins, and IR (10, 11, 16–18).

This study shows a significant positive correlation between 25(OH)VD and GSH status in adolescents. This led us to examine whether GSH regulates VD regulatory genes and 25(OH)VD status in the blood. Using the mouse model of 25(OH)VD deficiency, we show a significantly greater increase in blood 25(OH)VD levels after cosupplementation with VD (cholecalciferol)+LC (a GSH precursor) compared with results in a group supplemented with VD alone. In addition, supplementation with VD alone does not affect GSH, *TNF-α*, or IR levels in blood; however, when compared with results in the control group, cosupplementation using VD with LC significantly increases GSH levels and reduces oxidative stress, TNF-α, and IR levels in blood, and increases GSH levels and reduces oxidative stress in liver and muscle. Transfection studies demonstrate that GSH deficiency causes increased oxidative stress, downregulation of *VDBP*/VD-25-hydroxylase/*VDR*, and upregulation of *CYP24A1* in mouse hepatocytes and downregulation of *PGC-1α, VDR, GLUT4* in mouse myotubes, similar to results seen in HFD-fed mice.

Improvement in GSH status by LC prevented the downregulation of VD regulatory genes in hepatocytes and glucose metabolism genes in myotubes. These *in vitro* and *in vivo* studies demonstrate the dual roles of GSH in increasing circulating 25(OH)VD and augmenting the actions of active VD metabolites in one of the target tissues (skeletal muscle), which is a major site for glucose metabolism.

**FIG. 11.**
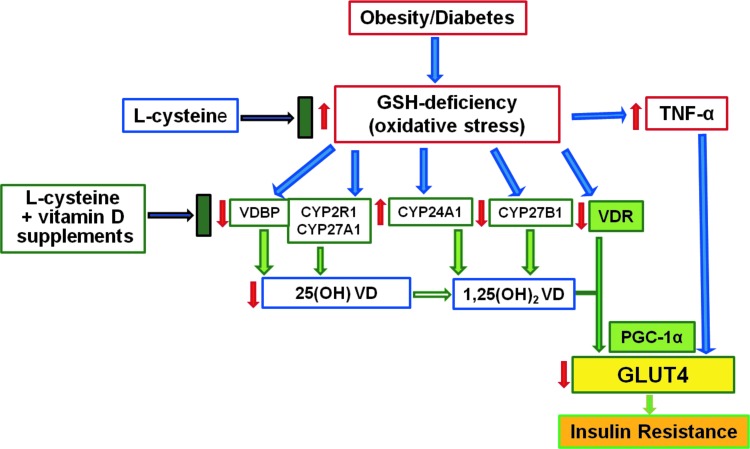
**Proposed mechanism for role of GSH deficiency in 25(OH)VD deficiency and potential of combined VD and LC supplementation on stimulation of VD regulatory genes and protection from 25(OH)VD deficiency and inflammation.**

Figure 11 outlines the proposed mechanism for role of GSH deficiency in 25(OH)VD deficiency and potential of combined VD and LC supplementation on stimulation of VD regulatory genes and protection from 25(OH)VD deficiency and inflammation. The mechanism potentially responsible for the increased blood levels of 25(OH)VD could be that LC upregulates the synthesis of GSH, thus improving the status of GSH, which reduces oxidative stress and prevents impaired (reduced) levels of *VDBP*/VD-25-hydroxylase/*VDR*, thereby helping protect the status of 25(OH)VD levels. VDBP is required for efficient transport and VD-25-hydroxylase is needed for the hydroxylation of cholecalciferol.

Furthermore, upregulation of VDR status in target tissues stimulates the translocation of the VDR/1,25(OH)_2_VD complex to the nucleus for activation of the *VDR/PGC-1α/GLUT-4* pathway responsible for metabolic actions of 1,25(OH)_2_VD. The *PGC-1α* is an inducible transcriptional coactivator that upregulates expression of *GLUT-4* in skeletal muscle and is a coactivator of the retinoic X receptor (*RXRα*) (40). *PGC-1α* functions as a cofactor for *NRF2*, which is implicated in the biosynthesis of GSH (24). Therefore, upregulation of *PGC-1*α and *GLUT-4* is beneficial in reducing IR and glycemia.

This study provides evidence for a previously undiscovered mechanism that describes how 25(OH)VD deficiency/inadequacy is linked to lower GSH levels and that boosting GSH status beneficially upregulates the genes of VD metabolism and *VDR*, both of which are needed to increase the bioavailability and blood levels of 25(OH)VD and reduce inflammation levels. GSH provides not only upregulation of VD regulatory genes but also adds to *GLUT-4* activation. Thus, increasing GSH status by combined supplementation with LC or a GSH precursor along with VD provides a novel approach to treat widespread 25(OH)VD deficiency/inadequacy in populations worldwide.

Investigation of mRNA expression and protein expression analyses of genes showed some differences between mRNA expression and protein expression levels. This could be due to variances in post-transcription regulation; differences in mRNA and protein turnover rates across the spectrum of genes involved; or differences in the technical precision of the methodology used. This study demonstrates that the improvement in the GSH status exerts measurable and beneficial effects on both mRNA and protein expression levels of *VDBP*/VD-25-hydroxylase/*VDR* as well as *PGC-1α/GLUT-4* genes. Blood levels of 25(OH)VD are lower in humans with a genetic mutation for VDBP or in VDBP-KD mouse models (19, 30, 52). Blood concentrations of VDBP are positively related to the half-life of circulating 25(OH)VD (30). This suggests that lower circulating 25(OH)VD levels could be a result of the decreased VDBP levels seen in obese adolescents. GSH is formed from LC by the enzymatic action of *glutamate-cysteine ligase* (*GCL*) and GSH synthetase (18). LC metabolism is facilitated by the LC transporter (24). Whether HFD feeding has any effect on the status of the LC transporter in tissues is not known.

Vitamin D helps the body absorb calcium and maintain bone and muscle health. Evidence in the literature supports the positive link between a high consumption of milk products and leafy vegetables and biomarkers of bone health and 25(OH)VD levels in the blood (50). In fact, milk and leafy vegetables are rich sources of both vitamin D and of GSH and methionine/LC. This may explain why consumption of food rich in LC/methionine and GSH can increase the bioavailability of VD and improve the quality of life. In addition, a large prospective E3N-Epic cohort study conducted among 64,233 middle-aged women reported that women consuming a diet with higher levels of total antioxidant capacity were found to have a lower risk for type 2 diabetes (35). Observations from the literature and our study suggest that there is an interaction between the consumption or status of the dietary nutrients LC/methionine and an enhanced bioavailability of vitamin D (cholecalciferol). The potential use of lower VD doses combined with a GSH precursor could provide a novel approach to correct 25(OH)VD deficiency/inadequacy. This research provides evidence for the recommendation to use a combination of VD and a GSH precursor for supplementation, rather than VD alone, to achieve greater success with the VD supplements widely used by the public in pursuit of better health.

These findings focus attention on the fact that the VD supplements widely consumed by the public are unlikely to be efficacious unless the status of the VD metabolism genes is improved by first correcting the status of GSH. This suggests that combined consumption of GSH precursors and VD, rather than solely using high-dose VD, is both novel and a better strategy with which to achieve a more efficacious bioavailability in response to cholecalciferol consumption and to increase blood levels of 25(OH)VD.

## Materials and Methods

### Enrollment of human subjects

This study was carried out after informed written consent was obtained from all subjects according to the protocol approved by the Louisiana State University Health Sciences Center (LSUHSC) Institutional Review Board (IRB). This study enrolled adolescent boys and girls ages 14–17 years, in good health (other than being overweight or IR), who provided written informed assent and parental consent. Inclusion criteria were children who are not smoking or taking any medicines, food supplements, or antihistamines. All subjects who gave written informed consent were invited to return to have blood drawn after fasting overnight (8 h). The exclusion criterion was that subjects were excluded if pregnant or suspected to be pregnant, applied only to female subjects. Urine was collected from the female subjects for urine pregnancy testing. Subjects were allowed to sit quietly for 10 min after which blood was collected from an easily accessible forearm vein using a butterfly needle and Vacutainer^®^ tube. Ethylenediaminetetraacetic acid (EDTA) blood tubes were brought to the research laboratory. Isolated plasma was stored in different aliquots and frozen immediately at −80°C for analyses of the biochemical parameters.

### Animal studies

Male C57BL/6J mice (5 weeks old, 20–24 g) were purchased from The Jackson Laboratory and acclimatized in the institutional animal house for 1 week. Mice were divided into various groups by computer-generated randomization and then housed and labeled in individual cages. They were fasted overnight and then weighed. Blood glucose was assessed by tail prick using an Accu-Chek glucometer (Boehringer Mannheim Corp., Indianapolis, IN). BW and blood glucose were monitored weekly. Control animals were fed a normal diet (lower in fat), while animals in the HFD group were fed HFD for 16 weeks. Flow diagram showing details of diet feeding of mice is given in Supplementary Figure S2. For studies with VD-deficient animals, the mice were maintained on a VD-deficient HFD for 16 weeks. After 8 weeks, the mice were supplemented by oral gavage for another 8 weeks with either 5 mg LC/kg BW daily (LC) or 67 IU VD/kg BW (+VD), or the same doses of cholecalciferol and l-cysteine (LC+VD) (Fig. 5).

In addition, two groups of mice maintained on the VD-deficient HFD were also simultaneously supplemented by oral gavage with either water or the same dose of the vehicle used for dissolving cholecalciferol (OO) (Fig. 5). The animals were maintained under standard housing conditions at 22°C ± 2°C with 12/12-h light/dark cycles. Normal diet, HFD, and VD-deficient HFD were purchased from The Jackson Laboratory and detailed composition of these diets is given in a recent publication (36). The amount of food intake was monitored at 12 and 16 weeks into the treatment period to assess consumption. At the end of 16 weeks, the animals were fasted overnight and then euthanized for analysis by exposure to isoflurane (Webster Veterinary Supply, Inc., Devens, MA). Blood was collected *via* heart puncture with a 19^1/2^-gauge needle into heparinized Vacutainer tubes. Plasma was isolated after centrifuging the blood in a 4°C centrifuge at 3000 rpm for 10 min. The livers were perfused with cold saline to free them of residual blood. Liver and gastrocnemius muscle were collected immediately, weighed, quickly diced, and frozen in liquid nitrogen at −80°C.

### Dose justification for LC and vitamin D

While LC can be taken as a supplement, it is also formed in the body from methionine. An adult ingests about 500 mg LC from dietary sources, assuming that an average protein intake is 90 g/day and that LC is about 0.6% of total protein (20). Similarly, taking into account daily dietary intake and diet composition, mice consume 3–5 mg LC/kg BW (32). The LC/methionine content of protein varies with the source of the protein. The LC dose used in our studies, 5 mg/kg BW, is theoretically a supplementation onefold to twofold that of the LC ordinarily consumed by the mice, which could be considered both modest and safe. The VD dose used was 67 IU/kg/day (1.67 μg/kg/day) as described in a recent publication (36). Cholecalciferol was dissolved in 0.1% OO and a stock solution of 1.67 μg/mL was prepared. An aliquot of 0.1 mL of the stock solution was given per 100 g BW using oral gavage on alternate days for 8 weeks. For alternate day gavaging, the supplementation dose was doubled to maintain a similar dose per day. The vehicle-OO control group is included in the treatment groups.

### Cell culture and treatment

FL83B mouse hepatocytes (ATCC^®^, Manassas, VA) were cultured and maintained in F-12K complete medium. Mouse C2C12 myoblasts were cultured at 37°C in an atmosphere of 5% CO_2_ in growth medium (GM) consisting of Dulbecco's modified Eagle's medium (DMEM) supplemented with heat-inactivated 10% fetal bovine serum and antibiotics (penicillin and streptomycin). Differentiation of myoblasts into myotubes was induced when the cells had achieved 90–95% confluence by switching the medium from GM to differentiation medium consisting of DMEM supplemented with 2% horse serum (5 days), then treated as described in the figures. siRNAs were purchased from Santa Cruz Biotechnology, Inc. (Dallas, TX), catalog numbers sc-41979 (GCLC), sc-36811 (VDR), and sc-38885 (PGC-1α). The control siRNA, a scrambled nonspecific RNA duplex that shares no sequence homology with any of the genes, was used as a negative control.

Cells were transiently transfected with 0–100 n*M* siRNA complex using Lipofectamine™2000 transfection reagent (Invitrogen, Carlsbad, CA) following the method described earlier (26, 43). The next day cells were treated with LC (0–300 μ*M*) for 6 h for LC-alone experiments. Pretreatment of the cells, maintained at a concentration of 1 × 10^6^/mL media, was done for 2 h with LC (0–300 μ*M*), followed by treatment for 22 h with cholecalciferol or 25(OH)VD (10 n*M*). *TNF-α* (0–250 pg/mL) was exposed for 6 h to differentiated myotubes.

### Justification for use of FL83B mouse hepatocytes and mouse C2C12 myoblasts

Liver is a major player in the synthesis and secretion of VDBP and the hydroxylation of cholecalciferol (vitamin D_3_) to 25-hydroxy-vitamin D. FL83B mouse hepatocytes express VDBP, CYP27A1, CYP27B1, CYP24A1, and VDR, but expression of CYP2R1 is very low and could not be accurately quantitated. However, we got reproducible gene analysis results for expression of VDBP, CYP27A1, CYP27B1, CYP24A1, and VDR genes using FL83B mouse hepatocytes. Liver contains both CYP27A1 and CYP2R1 and participates in the conversion of cholecalciferol (vitamin D_3_) to 25-hydroxy-vitamin D. The population studies have also shown a link between CYP2R1, GC (VDBP), CYP24A1, and VDR with that of circulating 25(OH)VD concentrations. Recent studies have shown a regulatory role of CYP27A1 gene expression on the blood concentrations of 25(OH)VD (4, 54, 57, 62, 63).

Thus, mouse hepatocytes used in this study have much strength and can be used for investigating the link between GSH-deficiency, oxidative stress, and VD regulatory genes. Muscle is a major site for glucose metabolism. Differentiation of myoblasts into myotubes is very reproducible and these cells express the glucose metabolism genes such as PGC-1α, RXRα, VDR, and GLUT-4. GLUT-4 is a master regulator for the maintenance of glucose metabolism. Thus, mouse C2C12 myoblasts have much strength to investigate the role of GSH deficiency in regulation of glucose metabolism pathways (42).

### Analysis of mRNA expression using quantitative polymerase chain reaction

Total RNA was extracted from cells or tissue using the TRIzol reagent (Life Technologies) following the manufacturer's instructions. The quality and quantity of the extracted RNA were determined on a NanoDrop spectrophotometer (Thermo Scientific). First-strand complementary DNA (cDNA) synthesis was performed using a commercially available High Capacity RNA-To-cDNA kit (Life Technologies) in a final reaction volume of 20 μL. Amplification of cDNA was performed on a 7900HT Real Time polymerase chain reaction (PCR) system (Applied Biosystems). PCR conditions were 2 min at 50°C, 10 min at 95°C, 40 cycles of 95°C for 15 s, and then 60°C for 60 s. Details of the TaqMan-FAM-labeled primer/probe used are given in Supplementary Table S4. Glyceraldehyde-3-phosphate dehydrogenase (GAPDH) was used as a housekeeping gene to normalize threshold cycle (CT) values.

To exclude nonspecific amplification and/or the formation of primer dimers, control reactions were performed in the absence of target cDNA. All of the experiments were run in triplicate. The relative amounts of mRNAs were calculated using the relative quantification (ΔΔCT) method. Supplementary Table S4 gives details of primer used in our studies.

### Western blot analysis

The tissue homogenates were processed for immunoblotting studies. To extract protein from liver and gastrocnemius muscle, ∼100 mg of tissue was homogenized in RIPA buffer on ice using a rotor/stator. RIPA buffer (50 m*M* Tris pH 8, 150 m*M* NaCl, 1% NP-40, 0.5% deoxycholic acid, and 0.1% SDS) was supplemented with protease and phosphatase inhibitors (1 m*M* phenylmethylsulfonyl fluoride, 5 μg/mL leupeptin, 2 μg/mL aprotinin, 1 m*M* EDTA, 10 m*M* NaF, and 1 m*M* NaVO4). Lysates were then centrifuged for 10 min at 10,000 *g* at 4°C. Supernatants were collected and the protein concentrations were determined using a BCA assay kit (Pierce/Thermo Scientific, Rockford, IL) for Western blot analysis and high performance liquid chromatography (HPLC) assay. Equal amounts (20 μg) of proteins were separated on 10% sodium dodecyl sulfate–polyacrylamide gel electrophoresis (SDS-PAGE) and transferred to a polyvinyl difluoride membrane. Membranes were blocked at room temperature for 2 h in a blocking buffer containing 1% bovine serum albumin to prevent nonspecific binding and then incubated with an appropriate primary antibody at 4°C overnight (26, 43).

The membranes were washed in TBS-T (50 m*M* Tris–HCl, pH 7.6, 150 m*M* NaCl, 0.1% Tween 20) for 30 min and incubated with an appropriate Horseradish peroxidase-conjugated secondary antibody (1:5000 dilution) for 2 h at room temperature. The protein bands were detected using ECL detection reagents (Thermo Scientific) and exposed on blue X-ray film (Phenix Research Products, Candler, NC). The technical replicates (*n* = 2) and biological replicates (*n* = 4) were done in all our immunoblot experiments. Western blot scans were analyzed using ImageJ software (developed by Wayne Rasband, National Institutes of Health, Bethesda, MD.* Densitometry analyses of Western blots were normalized with respect to β-actin or GAPDH (ratio).

### 25(OH)VD, 1,25(OH)_2_VD, VDBP, GSH, TNF-α, PTH, insulin, glucose, protein carbonyl, and MDA assays

Plasma levels of 25(OH) vitamin D were determined using an ELISA kit (Calbiotech, Spring Valley, CA) and 1,25(OH)_2_vitamin D using another ELISA kit (My BioSource, San Diego, CA). Plasma VDBP quantification was carried out using a kit purchased from ALPCO Diagnostics (Salem, NH). TNF-α was measured using an ELISA kit from R&D Systems (Minneapolis, MN). PTH (1–84) and insulin were determined using ELISA kits from ALPCO Diagnostics, and the HOMA-IR index was calculated (42). VDBP was measured using polyclonal antibodies (DRG Instruments, Springfield, NJ). The kit included polyclonal antibodies that detect total VDBP levels. In the ELISA, control samples were analyzed each time to check the variation from plate to plate on different days of analysis. Protocols as given in the manufacturer's instructions were followed using appropriate controls and standards.

Levels of GSH in plasma, tissues, and cultured cells were determined using HPLC (44). This assay determines total GSH status. Cell viability was determined using the Alamar Blue method (Alamar Biosciences, Sacramento, CA). Oxidative stress was assessed by the quantification of protein carbonyls and MDA using Protein Carbonyl Colorimetric and TBARS Assay Kits, respectively (Cayman Chemical, Ann Arbor, MI). Measurements of HbA_1c_, Complete Blood Count, glucose, and calcium were done at the clinical chemistry laboratories of LSUHSC-Shreveport. Due to limited amount of blood collected from each mouse, we borrowed diluents from the clinical laboratory and diluted the blood before taking it to the clinical laboratory, which reduced the amount of blood required for clinical tests. All chemicals were purchased from Sigma Chemical Co. (St. Louis, MO) unless otherwise mentioned.

### Statistical analysis

Data from clinical, cell culture, and mouse studies were analyzed using regression analyses and ANOVA with Sigma Stat software (SPSS, Chicago, IL). A *p*-value of ≤0.05 for a statistical test was considered significant.

### Data availability

The data supporting the findings are available within the article and the associated Supplementary Data of this study. Any other data are available from the corresponding author on reasonable request.

## Supplementary Material

Supplemental data

## Footnotes

^*^ http://rsb.info.nih.gov/ij/index.html

## Abbreviations Used

1,25(OH)_2_VD: 1alpha,25-dihydroxyvitamin D3
25(OH)VD: 25-hydroxyvitamin D
ANOVA: analysis of variance
BMI: body mass index
BW: body weight
cDNA: complementary DNA
CT: threshold cycle
CYP: cytochrome P450 enzymes
DMEM: Dulbecco's modified Eagle's medium
EDTA: ethylenediaminetetraacetic acid
ELISA: enzyme-linked immunosorbent assay
GAPDH: glyceraldehyde-3-phosphate dehydrogenase
GCL: glutamate-cysteine ligase
GCLC: glutamate-cysteine ligase catalytic subunit
GCLM: glutamate-cysteine ligase regulatory subunit
GLUT-4: glucose transporter type 4
GSH: glutathione
GM: growth medium
HFD: high-fat diet
HOMA: homeostatic model assessment
HPLC: high performance liquid chromatography
IR: insulin resistance
KD: knockdown
LC: L-cysteine
MDA: malondialdehyde
NRF2: nuclear factor erythroid-2-related factor
OO: olive oil
PCR: polymerase chain reaction
PGC-1α: peroxisome proliferator-activated receptor gamma coactivator 1-alpha
PTH: parathyroid hormone
RXRα: retinoic X receptor
TNF-α: tumor necrosis factor alpha
VD: vitamin D
VDBP: vitamin D binding protein
VDR: vitamin D receptor
