# Supplementary material for: Glutathione Stimulates Vitamin D Regulatory and Glucose-Metabolism Genes, Lowers Oxidative Stress and Inflammation, and Increases 25-Hydroxy-Vitamin D Levels in Blood: A Novel Approach to Treat 25-Hydroxyvitamin D Deficiency
Source: Antioxid Redox Signal. 2018 Oct 24;29(17):1792–807. doi: 10.1089/ars.2017.7462 (PMC6208166; doi:10.1089/ars.2017.7462)
Supplement: Supplemental data [file Supp_Data.pptx]

## Slide 1
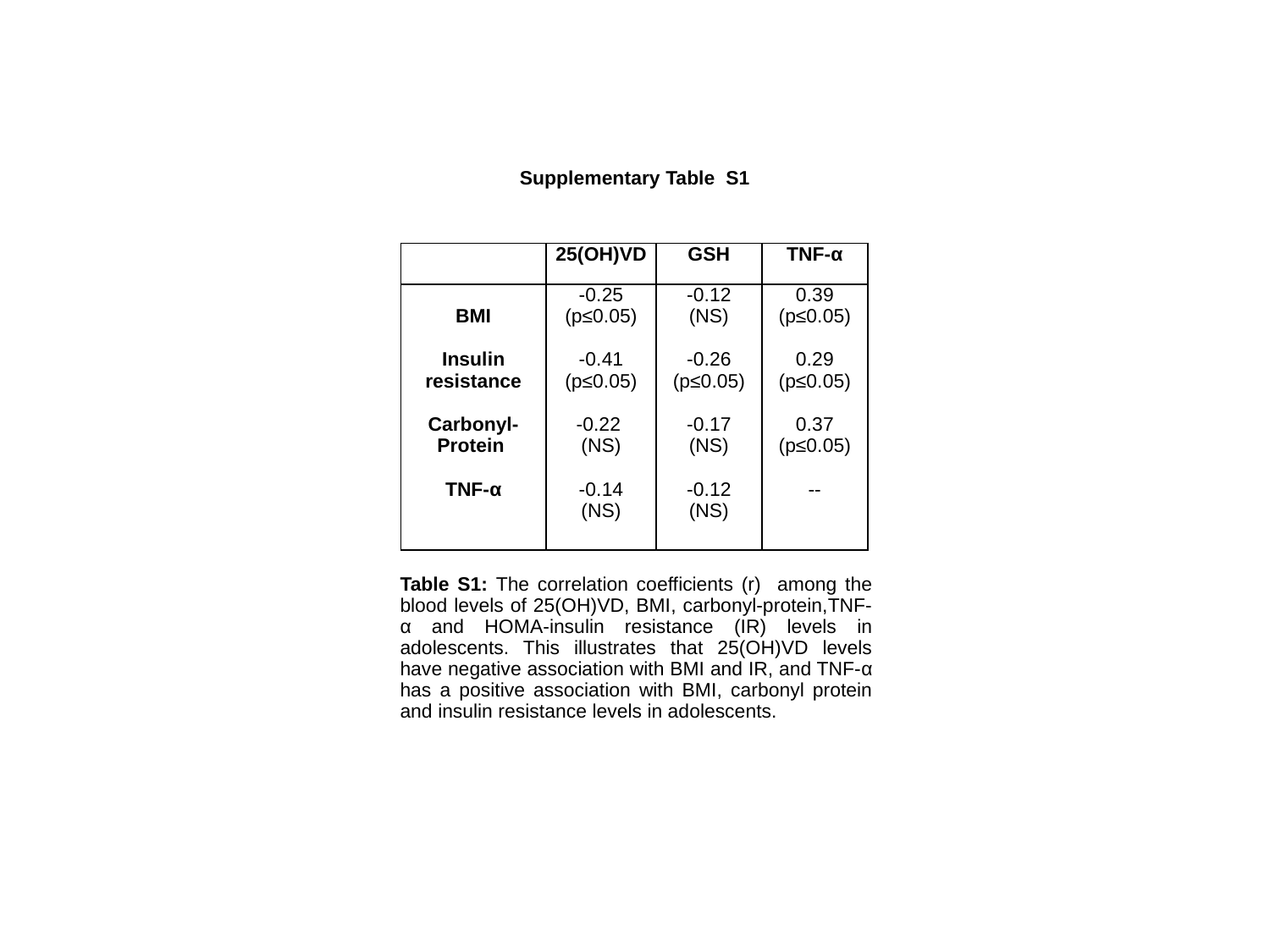

Supplementary Table S1
| | 25(OH)VD | GSH | TNF-α |
| --- | --- | --- | --- |
| BMI Insulin resistance Carbonyl- Protein TNF-α | -0.25 (p≤0.05) -0.41 (p≤0.05) -0.22 (NS) -0.14 (NS) | -0.12 (NS) -0.26 (p≤0.05) -0.17 (NS) -0.12 (NS) | 0.39 (p≤0.05) 0.29 (p≤0.05) 0.37 (p≤0.05) -- |
Table S1: The correlation coefficients (r) among the blood levels of 25(OH)VD, BMI, carbonyl-protein,TNF-α and HOMA-insulin resistance (IR) levels in adolescents. This illustrates that 25(OH)VD levels have negative association with BMI and IR, and TNF-α has a positive association with BMI, carbonyl protein and insulin resistance levels in adolescents.

## Slide 2
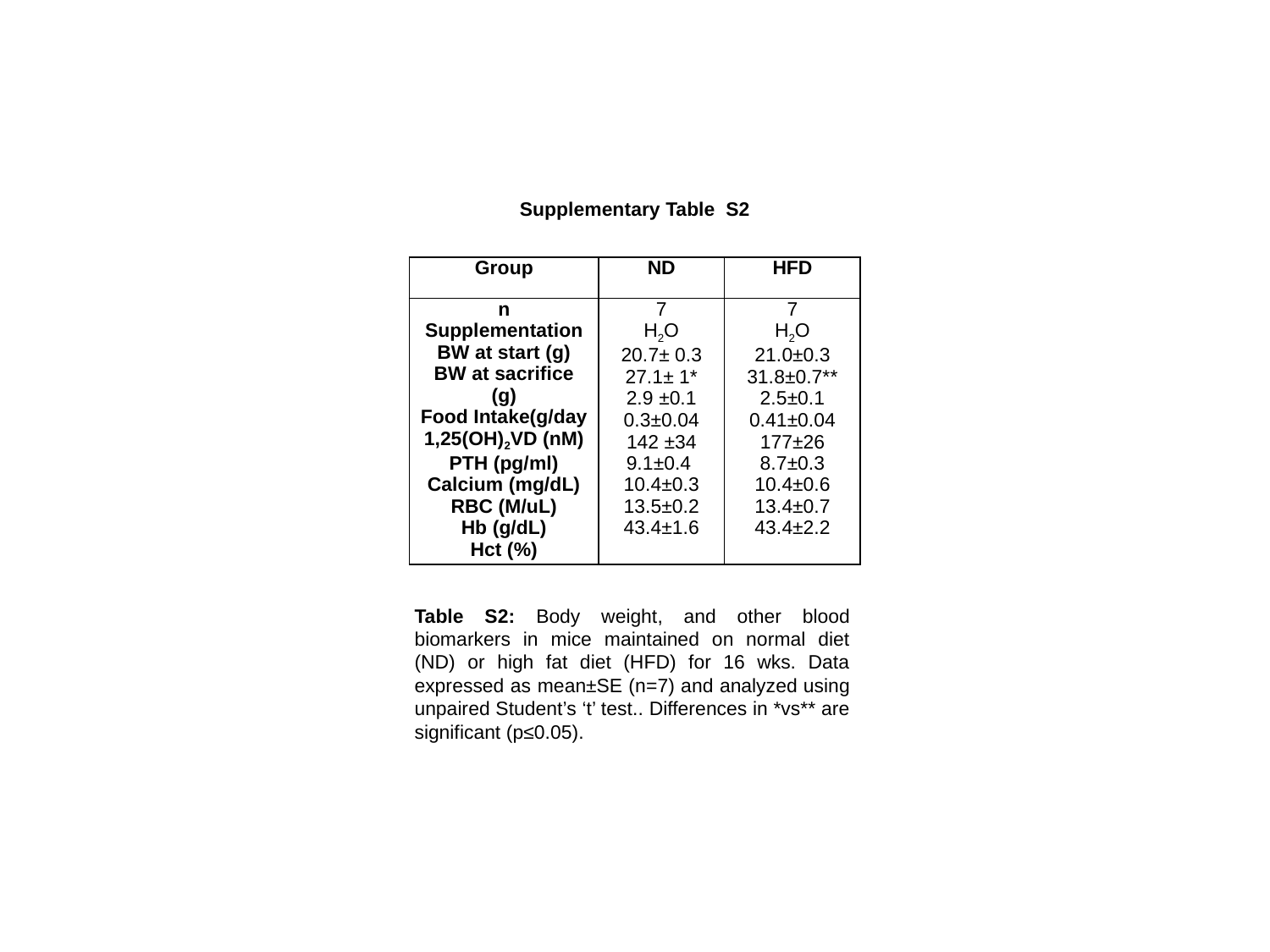

Supplementary Table S2
| Group | ND | HFD |
| --- | --- | --- |
| n Supplementation BW at start (g) BW at sacrifice (g) Food Intake(g/day 1,25(OH)2VD (nM) PTH (pg/ml) Calcium (mg/dL) RBC (M/uL) Hb (g/dL) Hct (%) | 7 H2O 20.7± 0.3 27.1± 1\* 2.9 ±0.1 0.3±0.04 142 ±34 9.1±0.4 10.4±0.3 13.5±0.2 43.4±1.6 | 7 H2O 21.0±0.3 31.8±0.7\*\* 2.5±0.1 0.41±0.04 177±26 8.7±0.3 10.4±0.6 13.4±0.7 43.4±2.2 |
Table S2: Body weight, and other blood biomarkers in mice maintained on normal diet (ND) or high fat diet (HFD) for 16 wks. Data expressed as mean±SE (n=7) and analyzed using unpaired Student’s ‘t’ test.. Differences in *vs** are significant (p≤0.05).

## Slide 3
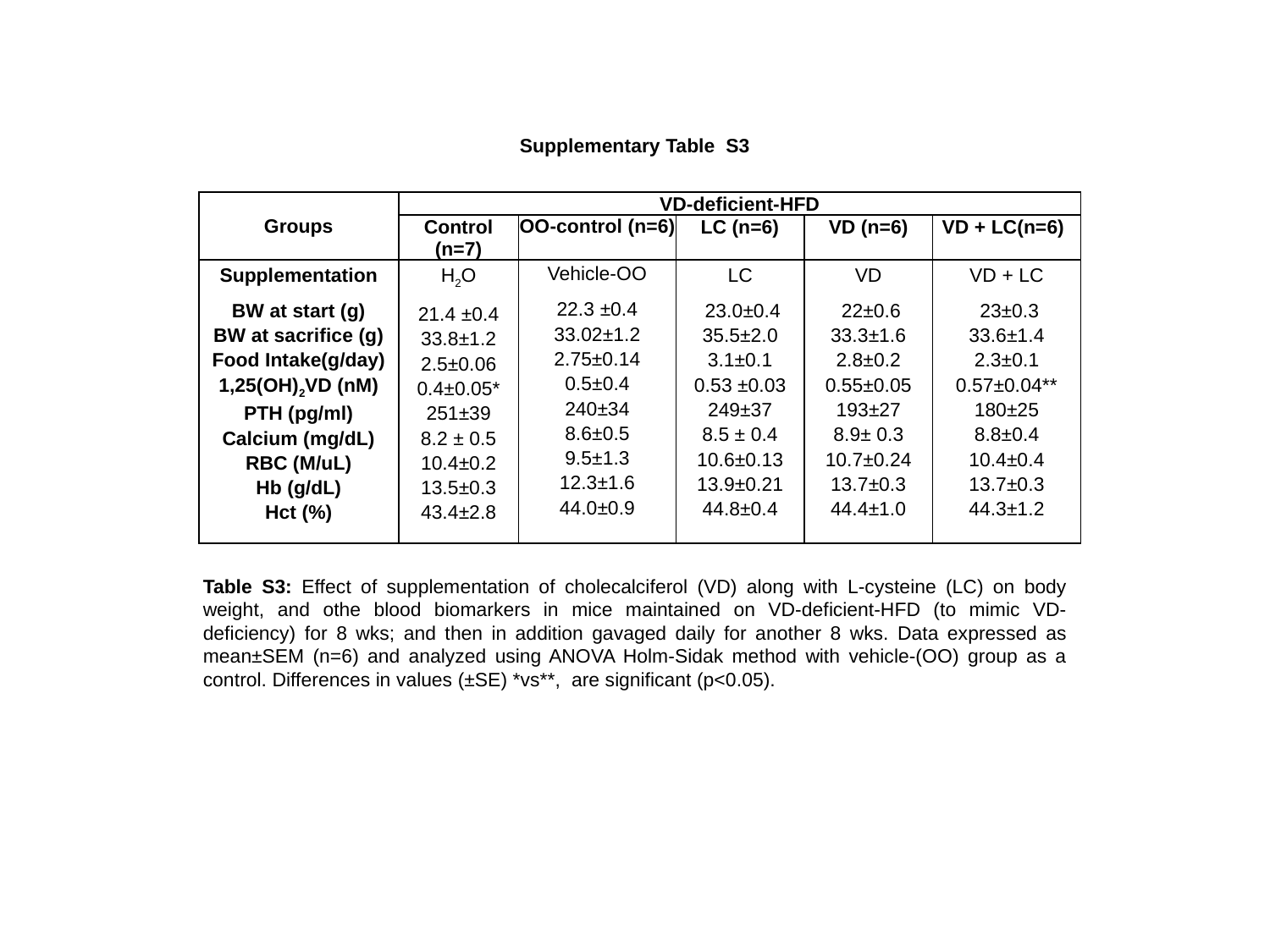

Supplementary Table S3
| Groups | VD-deficient-HFD | | | | |
| --- | --- | --- | --- | --- | --- |
| | Control (n=7) | OO-control (n=6) | LC (n=6) | VD (n=6) | VD + LC(n=6) |
| Supplementation   BW at start (g) BW at sacrifice (g) Food Intake(g/day) 1,25(OH)2VD (nM) PTH (pg/ml) Calcium (mg/dL) RBC (M/uL) Hb (g/dL) Hct (%) | H2O   21.4 ±0.4 33.8±1.2 2.5±0.06 0.4±0.05\* 251±39 8.2 ± 0.5 10.4±0.2 13.5±0.3 43.4±2.8 | Vehicle-OO   22.3 ±0.4 33.02±1.2 2.75±0.14 0.5±0.4 240±34 8.6±0.5 9.5±1.3 12.3±1.6 44.0±0.9 | LC    23.0±0.4 35.5±2.0 3.1±0.1 0.53 ±0.03 249±37 8.5 ± 0.4 10.6±0.13 13.9±0.21 44.8±0.4 | VD    22±0.6 33.3±1.6 2.8±0.2 0.55±0.05 193±27 8.9± 0.3 10.7±0.24 13.7±0.3 44.4±1.0 | VD + LC    23±0.3 33.6±1.4 2.3±0.1 0.57±0.04\*\* 180±25 8.8±0.4 10.4±0.4 13.7±0.3 44.3±1.2 |
Table S3: Effect of supplementation of cholecalciferol (VD) along with L-cysteine (LC) on body weight, and othe blood biomarkers in mice maintained on VD-deficient-HFD (to mimic VD-deficiency) for 8 wks; and then in addition gavaged daily for another 8 wks. Data expressed as mean±SEM (n=6) and analyzed using ANOVA Holm-Sidak method with vehicle-(OO) group as a control. Differences in values (±SE) *vs**, are significant (p<0.05).

## Slide 4
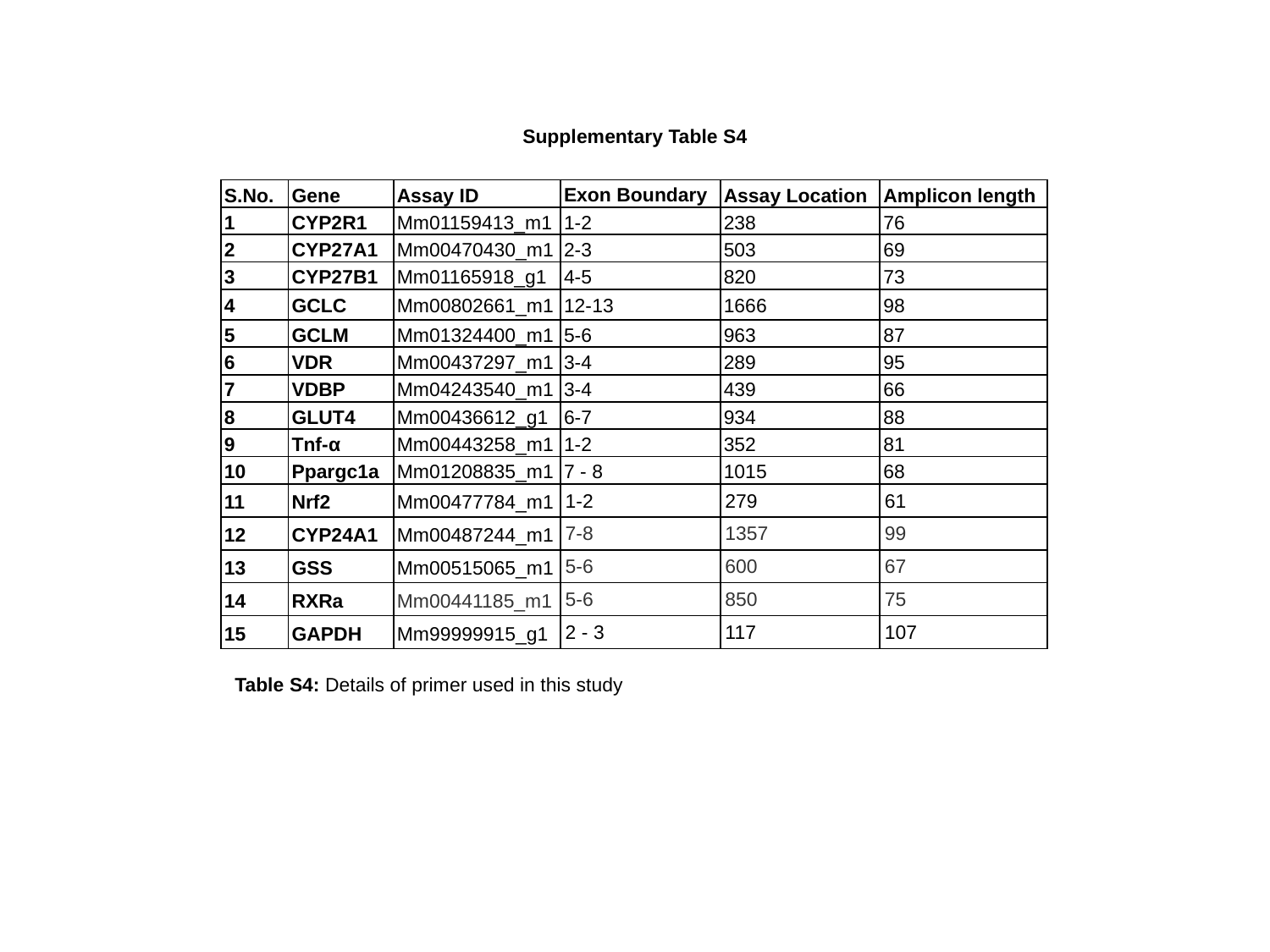

Supplementary Table S4
| S.No. | Gene | Assay ID | Exon Boundary | Assay Location | Amplicon length |
| --- | --- | --- | --- | --- | --- |
| 1 | CYP2R1 | Mm01159413\_m1 | 1-2 | 238 | 76 |
| 2 | CYP27A1 | Mm00470430\_m1 | 2-3 | 503 | 69 |
| 3 | CYP27B1 | Mm01165918\_g1 | 4-5 | 820 | 73 |
| 4 | GCLC | Mm00802661\_m1 | 12-13 | 1666 | 98 |
| 5 | GCLM | Mm01324400\_m1 | 5-6 | 963 | 87 |
| 6 | VDR | Mm00437297\_m1 | 3-4 | 289 | 95 |
| 7 | VDBP | Mm04243540\_m1 | 3-4 | 439 | 66 |
| 8 | GLUT4 | Mm00436612\_g1 | 6-7 | 934 | 88 |
| 9 | Tnf-α | Mm00443258\_m1 | 1-2 | 352 | 81 |
| 10 | Ppargc1a | Mm01208835\_m1 | 7 - 8 | 1015 | 68 |
| 11 | Nrf2 | Mm00477784\_m1 | 1-2 | 279 | 61 |
| 12 | CYP24A1 | Mm00487244\_m1 | 7-8 | 1357 | 99 |
| 13 | GSS | Mm00515065\_m1 | 5-6 | 600 | 67 |
| 14 | RXRa | Mm00441185\_m1 | 5-6 | 850 | 75 |
| 15 | GAPDH | Mm99999915\_g1 | 2 - 3 | 117 | 107 |
Table S4: Details of primer used in this study

## Slide 5
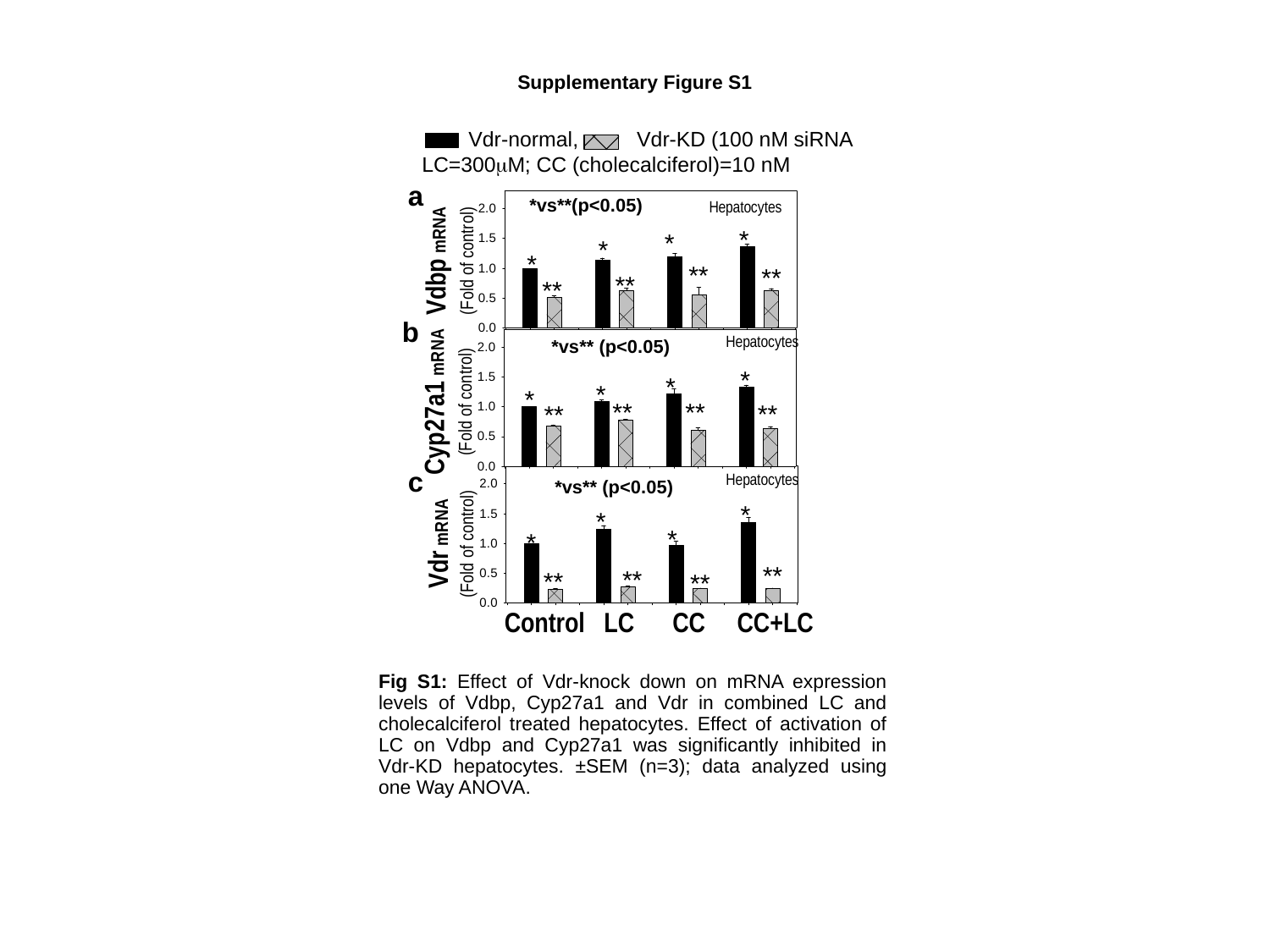

Supplementary Figure S1
Fig S1: Effect of Vdr-knock down on mRNA expression levels of Vdbp, Cyp27a1 and Vdr in combined LC and cholecalciferol treated hepatocytes. Effect of activation of LC on Vdbp and Cyp27a1 was significantly inhibited in Vdr-KD hepatocytes. ±SEM (n=3); data analyzed using one Way ANOVA.

## Slide 6
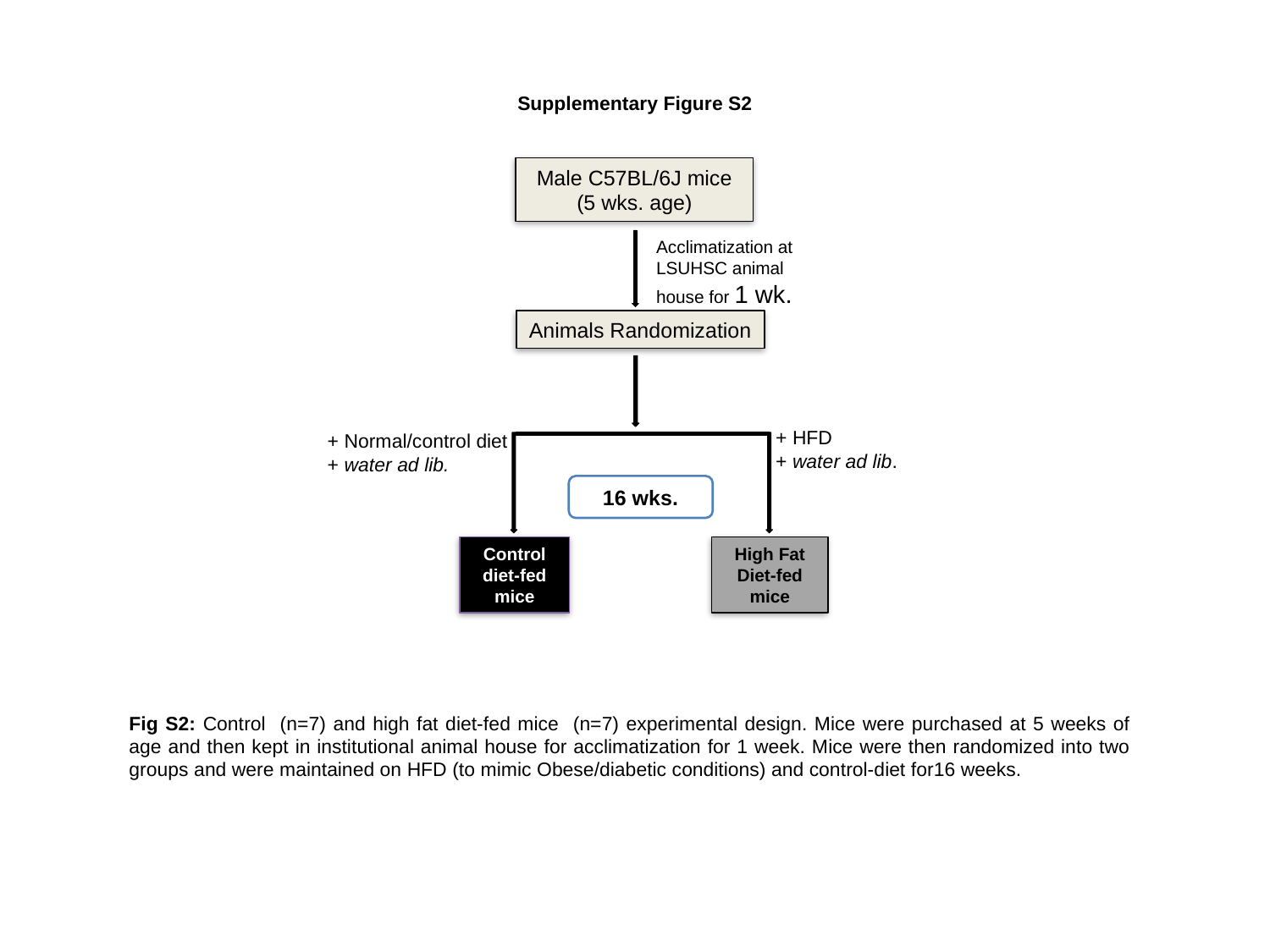

Supplementary Figure S2
Male C57BL/6J mice (5 wks. age)
Acclimatization at
LSUHSC animal
house for 1 wk.
Animals Randomization
+ HFD
+ water ad lib.
+ Normal/control diet
+ water ad lib.
16 wks.
Control diet-fed mice
High Fat Diet-fed mice
Fig S2: Control (n=7) and high fat diet-fed mice (n=7) experimental design. Mice were purchased at 5 weeks of age and then kept in institutional animal house for acclimatization for 1 week. Mice were then randomized into two groups and were maintained on HFD (to mimic Obese/diabetic conditions) and control-diet for16 weeks.
